# Economic Analysis of Low Volume Interventions Using Real-World Data: Costs of HIV Self-Testing Distribution and HIV Testing Services in West Africa From the ATLAS Project

**DOI:** 10.3389/frhs.2022.886513

**Published:** 2022-06-27

**Authors:** Métogara Mohamed Traore, Kéba Badiane, Anthony Vautier, Arlette Simo Fotso, Odé Kanku Kabemba, Nicolas Rouveau, Mathieu Maheu-Giroux, Marie-Claude Boily, Joseph Larmarange, Fern Terris-Prestholt, Marc d'Elbée

**Affiliations:** ^1^Solidarité Thérapeutique et Initiative pour la Santé, Abidjan, Côte d'Ivoire; ^2^Solidarité Thérapeutique et Initiative pour la Santé, Dakar, Senegal; ^3^Centre Population et Développement (Ceped), Institut de Recherche pour le Développement (IRD), Université de Paris, Inserm, Paris, France; ^4^Solidarité Thérapeutique et Initiative pour la Santé, Bamako, Mali; ^5^Department of Epidemiology, Biostatistics, and Occupational Health, School of Population and Global Health, McGill University, Montréal, QC, Canada; ^6^Department of Infectious Disease Epidemiology, Medical Research Council Centre for Global Infectious Disease Analysis, Imperial College London, London, United Kingdom; ^7^Department of Global Health and Development, Faculty of Public Health and Policy, London School of Hygiene and Tropical Medicine, London, United Kingdom; ^8^Department of Global Health in the Global South, Bordeaux Population Health, Institut National de la Santé et de la Recherche Médicale (Inserm), University of Bordeaux, Bordeaux, France

**Keywords:** real-world data, HIV self-testing, HIV testing services, health facilities, cost analysis, Côte d'Ivoire, Mali, Senegal

## Abstract

Achieving the first 95 of the UNAIDS targets requires the implementation of innovative approaches to knowing one's HIV status. Among these innovations is the provision of HIV self-testing (HIVST) kits in west Africa by the international partner organization Solthis (IPO). In order to provide guidance for the optimal use of financial resources, this study aims to estimate the program and site level costs of dispensing HIVST as well as HIV testing services (HTS)-excluding HIVST-in health facilities in Côte d'Ivoire, Mali and Senegal as part of the ATLAS project. We estimated from the provider's perspective, HIVST and HTS incremental costs using top-down and bottom-up costing approaches and conducted a time and motion study. We identified costs at the *program level* for HIVST (including IPO central costs) and at the *site level* for HIVST and HTS. The economic costs of distributing HIVST kits were assessed in 37 health facilities between July 2019 and March 2021 (21 months). Sensitivity analyses were also performed on unit costs to examine the robustness of our estimates related to key assumptions. In total, 16,001 HIVST kits were dispensed for 32,194 HTS sessions carried out. Program level HIVST average costs ranged $12–286, whereas site level costs ranged $4–26 across distribution channels and countries. Site level HTS costs ranged $7–8 per testing session, and ranged $72–705 per HIV diagnosis. Across countries and channels, HIVST costs were driven by personnel (27–68%) and HIVST kits (32–73%) costs. The drivers of HTS costs were personnel costs ranging between 65 and 71% of total costs across distribution channels and countries, followed by supplies costs between 21 and 30%. While program level HIVST average costs were high, site level HIVST average costs remained comparable to HTS costs in all countries. Health facility-based distribution channels operating at low volume exhibit high proportion of central costs which should be considered carefully for financial planning when run alongside high volumes mobile outreach distribution channels. HIVST can diversify the HIV testing offer at health facilities, thus improving access to screening for target populations not reached by HTS services.

## Introduction

The ambitious 95-95-95 strategy was announced by UNAIDS in 2020 aiming to end the AIDS epidemic by 2030 (1) by achieving 95% diagnosed among all people living with HIV (PLHIV), 95% on antiretroviral therapy (ART) among diagnosed, and 95% virally suppressed among treated (2). It is the result of the programmatic efforts of the actors who have thus made it possible to double in more ten years the annual volume of HIV tests carried out in sub-Saharan Africa (3, 4). In 2021, key populations and their sexual partners accounted for 72% of new adult HIV infections, and women and girls (aged 15 to 49 years) represented 65% in west and central Africa (5). The prevalence in the general population (15–49) was ranging from 0.6 to 3.8 (Côte d'Ivoire = 3.8; Mali = 1.3; Senegal = 0.6) (5). In west and central Africa, the coverage of HIV testing and antiretroviral therapy has grown at a quicker pace in recent years (81% people living with HIV who know their status), with nearly 77% of people living with HIV receiving antiretroviral therapy and 62% virally suppressed (5). In 2020, key populations (sex workers and their clients, gay men and other men who have sex with men, people who inject drugs, transgender people) and their sexual partners accounted for 65% of HIV infections globally whose 39% of new HIV infections in sub-Saharan Africa. Also, the risk of contracting HIV is, respectively, 35, 26, and 25 times higher among people who inject drugs, for sex workers and among gay men and other men who have sex with men (6).

In order to reach the first 95 in west Africa, it is necessary to implement approaches that reach those in need of HIV testing and who are being missed (4). One of the means used for this strategy is the provision of HIV self-testing (HIVST) kits to populations at risk. HIVST is an innovative method where a person collects his/her own specimen (oral fluid or blood), performs a rapid HIV test, and interprets the result, often in a private environment (7). It is a complementary testing approach, often seen as convenient and is highly accurate (8–10). Recent studies evaluated opportunities for implementing HIVST among key populations in Ghana (11). The authors found that a conducive and private environment was a factor facilitating the learning and practice of HIVST. It reduced stigma, and guaranteed the safety of vulnerable people by contributing to high rates of HIVST use. Other studies suggested that the ability of primary contacts to distribute HIVST kits to their contacts could be limited due to specific barriers such as acts of violence against FSW by their clients (12, 13).

The ATLAS project, implemented by the international partner organization (IPO) Solthis (Solidarité thérapeutique et initiative pour la santé) in Côte d'Ivoire, Mali and Senegal, promotes the distribution of HIVST to hard-to-reach/hidden populations as a complementary approach to traditional HIV testing services (HTS). In the context of this study, HTS definition excludes HIVST kits provision. Two main strategies are adopted: a large-scale strategy using mobile outreaches (mobile strategy) and a small-scale strategy in health facilities. The health facilities strategy aims to reach female sex workers (FSW), men who have sex with men (MSM), people who use drugs (PWUD), partners of sexually transmitted infections patients (STI) and sexual partners of PLHIV (Index Testing) in health facilities (Appendix 1). Regarding the introduction of HIVST in health facilities (fixed strategies), HTS is preferred (except PLHIV) for the patient coming in the health facility and HIVST is only offered for patients refusing HTS. In a primary distribution strategy, the HIVST kit is distributed directly to the intended user whereas for the secondary distribution, the kit is offered to a primary contact who will redistribute it to a secondary recipient. Secondary distribution strategies is preferred with the ATLAS project, allowing to reach hidden key populations, such as clients of FSW, partners of MSM, or index partners of pregnant women who are not routinely tested (12–15). Large scale programs being expanded nationally often benefit from economy of scale, even when incurring high initial fixed costs during the pilot phases as observed with ATLAS mobile strategies presented elsewhere (16, 17). On the other hand, low-volume interventions such as ATLAS health facilities strategies often pose challenges to estimating average costs (=total program costs/number of HIVST kits distributed) for informing analyses of cost-effectiveness and national program budgets. These observed average costs tend to be very high due to low output volumes and need to be considered carefully if to be used by program planners and researchers.

To establish the merits of the use of HIVST and therefore the adaptation of programmatic strategies and optimal financing in the fight against HIV in health facilities, data on costs at the level of programs and intervention sites prove necessary (18). Therefore, there is an urgent need to fill this knowledge gap regarding the costs of integrating HIVST into low-volume structures in West Africa.

The objective of this study is to estimate and analyse the program and site level costs of dispensing HIVST and providing HTS in health facilities using real-word data collected in Côte d'Ivoire, Senegal and Mali as part of the ATLAS project. The results of this study will inform HIV funding programs while providing key information for guiding actions tailored to low-volume health facilities.

## Materials and Methods

### Cost and Programmatic Data Collection

This study focused on HIVST distribution channels implemented in health facilities and targeting, FSW, MSM, PWUD, partners of STI patients and partners of PLHIV (Table 1). There were no MSM distribution channel in Senegal, and no PWUD channel in Mali.

**Table 1:** Overview of the ATLAS project's implementing health facilities by country.

**Characteristics**	**Côte d'Ivoire**	**Mali**	**Senegal**
**Analysis period**	**October 19 to March 21**	**July 19 to** **March 21**	**July 19 to** **March 21**
**Number of sites per distribution channel**			
FSW	3	4	1
MSM	3	4	0
PWUD	1	0	1
STI	10	5	5
Index testing	12	8	3
Health facilities	16	11	10
Number of trained HIVST providers	123	50	35
Number of trained HTS providers	77	56	50
Number of regions sampled/Total number of regions in country	4/33	4/10	3/14
Number of districts covered/Total Number of districts in country	6/113	6/65	9/79

*HIVST, HIV Self-Testing kit; HTS, HIV Testing Services; FSW, Female Sex workers; MSM, Men who have Sex with Men; PWUD, People who use drugs; STI, sexually transmitted infections; PLHIV, people living with HIV; Index Testing, sexual partners of PLHIV*.

For HIVST cost data collection in the *real-word*, we used a ***top-down costing*** approach to collate the IPO's financial expenditures and categorize each cost item by input type and distribution channel (19). Also, on site observations were conducted to estimate donated goods/services such as personnel in the field or some equipment costs ***(bottom-up costing)*** to estimate economic costs (Appendix 2). Finally, a ***time and motion study*** was conducted to allocate field-based personnel costs based on the proportion of time spent on HIVST distribution and HTS, respectively (18, 20). HIVST program data were obtained from the IPO. HTS cost and program data were collected through bottom-up costing only.

We conducted convenience sampling of sites, based on the type of distribution channel, the cohort size estimated of each distribution channel, and contextual factors such as accessibility to the HIV testing site. Because HIVST implementation was progressive over time, the analysis period was 21 months in Senegal and Mali, and 18 months in Côte d'Ivoire. Some sites having opened in 2021, only health facilities where the distribution of HIVST was effective throughout the implementation phase were taken into account and started in 2019 were retained. There were 16 distribution sites in Côte d'Ivoire, 10 in Mali, and 11 in Senegal. The majority of these sites were public facilities (78%). A site is defined either as a primary health care facility for general population and/or key populations, a sexual health clinic, or a community health centre recognized by government authorities. Overall, HIVST kits were distributed by 208 agents (Côte d'Ivoire = 123; Mali = 50; Senegal = 35) of whom 183 were counselors (Côte d'Ivoire = 77; Mali = 56; Senegal = 50) also providing HTS. The study distinguishes providers that are the health care workers paid by the state budget (doctors, nurses, midwives, laboratory technicians or social workers) and financial and technical partners that are peer educators involved in project activities.

### Analytical Approach

Data were analyzed among the sample sites according to the distribution channels FSW (Côte d'Ivoire = 3; Mali = 4; Senegal = 1), MSM (Côte d'Ivoire = 3; Mali = 4), PWUD (Côte d'Ivoire = 1; Senegal = 1), STI (Côte d'Ivoire = 10; Mali = 5; Senegal = 5) and Index Testing (Côte d'Ivoire = 12; Mali = 8; Senegal = 3) and we used standardized costing guidelines and analyzed data, ensuring consistency of methods across countries (16, 21). We conducted two economic cost analyses from the *provider's perspective*: HIVST and HTS. We distinguished between ***program*** and ***site level costs*** (Appendix 2).

**Figure 1:**
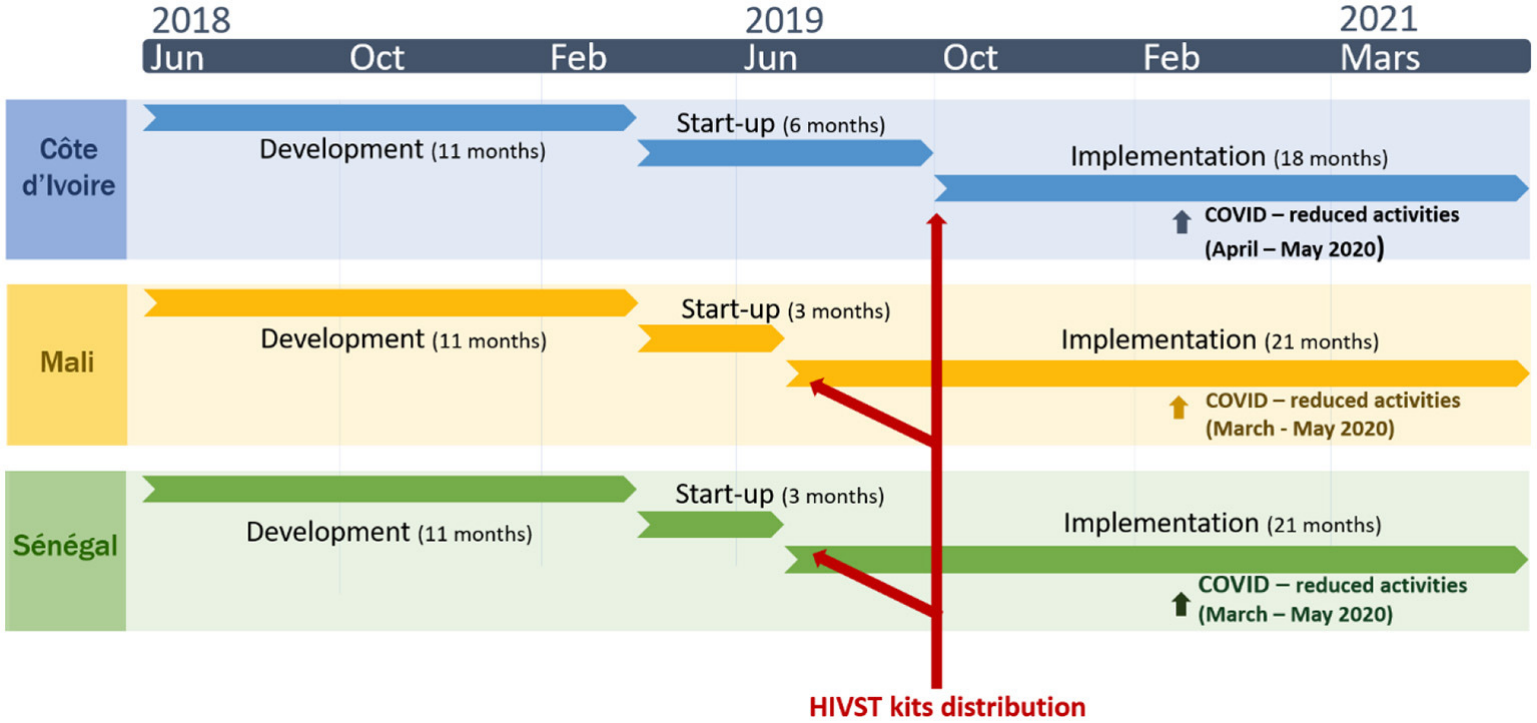
Description of the ATLAS project's three HIV self-testing (HIVST) deployment phases in Côte d'Ivoire, Senegal, and Mali over 2018–2021 [Source: (16)].

***Program costs*** were estimated for HIVST only, and include all costs incurred during the project phases and at all intervention levels (central, implementing partners headquarters, and delivery site levels) and provide costs of the entire program. These costs are distributed according to the three intervention phases of the project *(Development, Start-up (start-up and other costs), Implementation)* (Figure 1). The ***development phase*** (June 2018-March 2019) identified sustainable distribution models in each country; the ***start-up phase*** [April 2019-July 2019 (Senegal/Mali), October 2019 (Côte d'Ivoire)] consisted of the validation, adaptation and implementation of the training strategy for HIVST providers; and the ***implementation phase*** (until March 2021) corresponded to the effective distribution of HIVST kits, supervision and data validation activities. For HIVST, we also estimated ***site level costs*** at health facilities, therefore excluding central costs (IPO costs). For HTS, above site-level costs were not estimated because we did not have data access, therefore, only site level costs were estimated.

The HIVST kit purchase cost was provided by the IPO at $2.63 for Côte d'Ivoire and $3.08 for Senegal and Mali. Start-up, training, and all other capital costs were annualized using a 3% discount rate. All costs were estimated in 2021 US dollars (US$) using annual exchange rates (22). Inputs were categorized into capital and recurrent costs. We evaluated total costs, average and median costs per HIVST kit distributed, per HTS patient tested, and per HIV diagnosis.

Financial costs were allocated to sites based on allocation factors presented in Appendix 3. These factors included the percentage of HIVST distributors in each site, the estimated cohort size estimated of each distribution channel, the percentage of HIVST kits distributed, and the percentage of expenditure. The percentage of expenditure was an average obtained by weighting the total expenditures after all other allocation factors had been applied to relevant cost inputs. For the estimation of the HIVST delivery costs, we conducted an incremental cost analysis, where only additional resources needed to add HIVST onto existing service provision were considered. The values of the “average costs” in the tables are based on an average of unit costs (total costs/output) from each site per channel for each country.

Finally, we conducted a series of one-way *sensitivity analyses* to examine the robustness of the results to different assumptions used in the cost analysis as follows (23, 24). We assessed the influence of (1) the allocation factors by mutually inverting the number of distributors by patient cohort for each distribution channel; (2) the duration of the project by varying the number of year used to annualise of the life of the development phases (5 and 15 years, base of 10 years), the duration of start-ups (2.5 and 7.5 years, base of 5 years) and training and awareness costs (1 and 3 years, base of 2 years); (3) the discount rate by the varying the annualization rate for all costs combined from 0 to 16% (compared to base case: 3%) (21).

## Results

### Presentation of Program Level HIV Self-Testing Costs

**Figure 2:**
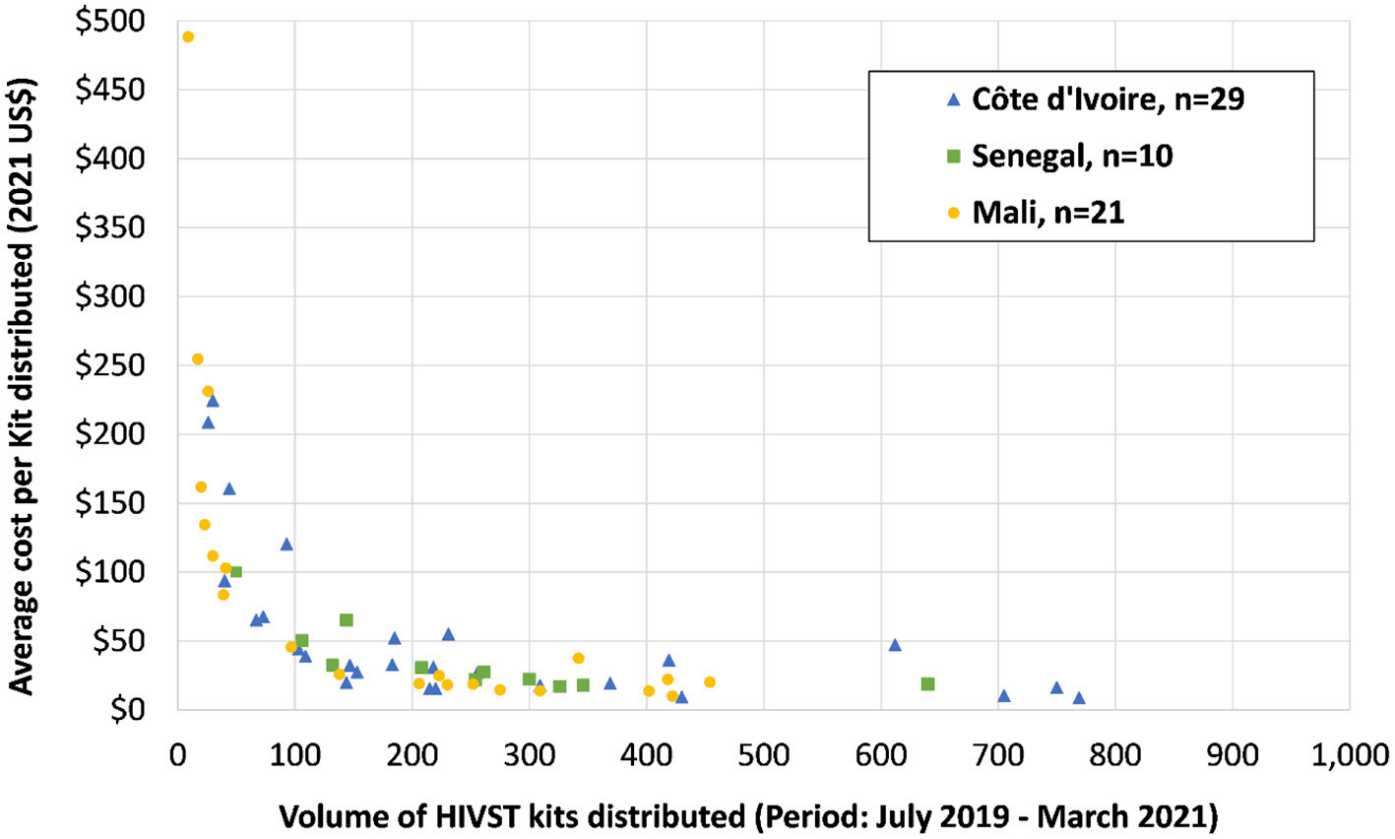
Average costs in health facilities by volume of HIVST Kits distributed by country (in 2021 US$).

Figure 2 presents the average cost per HIVST kit distributed vs. the volume of kits distributed for each site. The greater the volume of kits distributed, the lower is the average cost of the HIVST kit and vice versa, in all three countries.

Table 2 shows that in Côte d'Ivoire, the average costs in health facilities per HIVST kit distributed varied between distribution channels from $15 to 93 (FSW: $15; MSM: $15; PWUD: $12; STI: $31; Index Testing: $91), average costs per distributed kit ranged from $20 to 286 (FSW: $19; MSM: $90; STI: $145; Index: $286) in Mali, and ranged between $19 and 38 (FSW: $22; PWUD: $19; STI: $30; Index Testing: $38) in Senegal.

**Table 2:** Total, average, median and range of program level HIV self-testing costs, by country, by distribution channel, and by intervention phase in health facilities (in 2021 US$).

	**Côte d'Ivoire**									
	**FSW**		**MSM**		**PWUD**		**STI**		**Index testing**	
	**$**	**%**	**$**	**%**	**$**	**%**	**$**	**%**	**$**	**%**
Number of sites	3		3		1		10		12	
**Intervention Phases**										
Development	288	*2%*	286	*2%*	138	*1%*	1,627	*3%*	3,349	*3%*
Start-up (start-up and other costs)	2,905	*20%*	1,250	*10%*	1,775	*13%*	13,701	*23%*	32,101	*24%*
Implementation	11,304	*78%*	11,316	*88%*	11,290	*86%*	44,599	*74%*	96,342	*73%*
Total costs	14,498	*100%*	12,852	*100%*	13,203	*100%*	59,928	*100%*	131,793	*100%*
HIVST kits distributed	1,019		1,128		1,117		2,782		3,260	
Average cost per HIVST kit distributed	14.65		14.64		11.82		31.18		92.62	
Median of the average cost per HIVST kit distributed per site	15.38		15.42		n.a.		29.75		61.27	
Range (min–max)	(9–19)		(9–20)		n.a.		(10–65)		(15–224)	
	**Mali**									
	**FSW**		**MSM**		**STI**		**Index testing**			
	**$**	**%**	**$**	**%**	**$**	**%**	**$**	**%**		
Number of sites	*4*		*4*		*5*		*8*			
**Intervention phases**										
Development	601	*3%*	597	*4%*	895	*4%*	1,250	*3%*		
Start-up (start-up and other costs)	6,401	*30%*	2,115	*14%*	4,053	*18%*	22,875	*47%*		
Implementation	14,154	*67%*	12,182	*82%*	17,718	*78%*	24,615	*51%*		
Total costs	21,156	*100%*	14,894	*100%*	22,666	*100%*	48,740	*100%*		
HIVST kits distributed	1,131		516		948		1,378		
Average cost per HIVST kit distributed	19.51		89.73		124.96		98.83			
Median of the average cost per HIVST kit distributed per site	19.15		107.26		24.77		41.51			
Range (min–max)	(14–26)		(10–134)		(14–488)		(19–255)			
	**Senegal**									
	**FSW**		**PWUD**		**STI**		**Index testing**			
	**$**	**%**	**$**	**%**	**$**	**%**	**$**	**%**		
Number of sites	1		1		5		3			
**Intervention phases**										
Development	400	*6%*	881	*7%*	1,864	*6%*	868	*5%*		
Start-up (start-up and other costs)	2,015	*30%*	1,666	*14%*	13,250	*39%*	6,485	*41%*		
Implementation	4,266	*64%*	9,483	*79%*	18,691	*55%*	8,647	*54%*		
Total costs	6,681	*100%*	12,030	*100%*	33,805	*100%*	16,000	*100%*		
HIVST kits distributed	300		640		1,331		451		
Average cost per HIVST kit distributed	22.27		18.8		29.85		37.81			
Median of the average cost per HIVST kit distributed per site	n.a.		n.a.		21.88		32.39			
Range (min–max)	n.a.		n.a.		(17–65)		(31–50)			

*HIVST, HIV Self-Testing kit; FSW, Female Sex workers; MSM, Men who have Sex with Men; PWUD, People who use drugs; STI, sexually transmitted infections; Index Testing, sexual partners of PLHIV; PLHIV, people living with HIV; n.a., not applicable*.

### Presentation of Program and Site Level HIV Self-Testing Costs and Site Level HTS Costs

**Figure 3:**
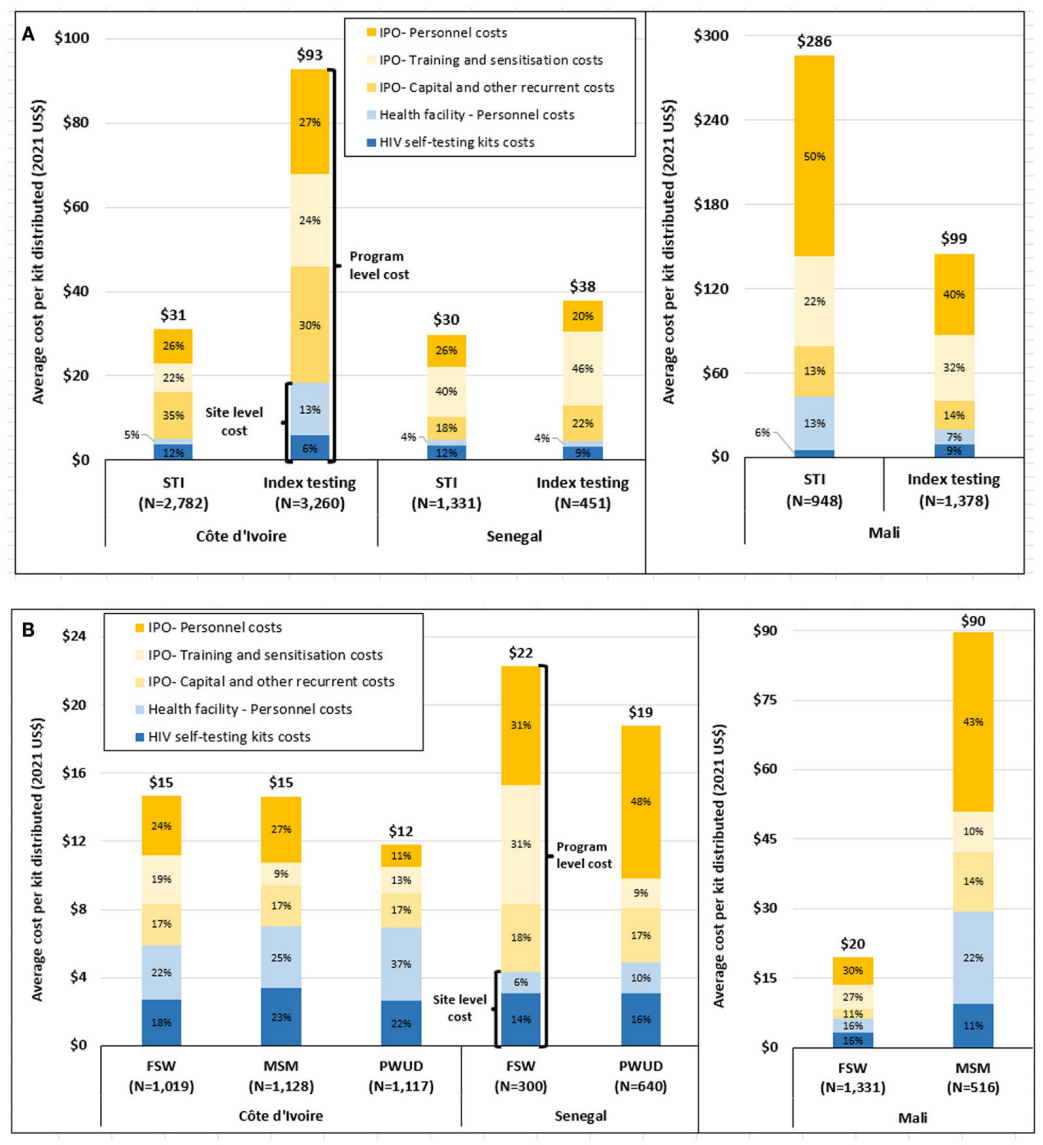
**(A)** Program and site level HIVST average costs in health facilities for the STI and Index testing distribution channels by country (in 2021 US$). **(B)** Program and site level HIVST average costs health facilities for the FSW, MSM and PWUD distribution channels by country (in 2021 US$). HIVST, HIV Self-Testing kit; FSW, Female Sex workers; MSM, Men who have Sex with Men; PWUD, People who use drugs; STI, sexually transmitted infections; Index, sexual partners of PLHIV; PLHIV people living with HIV.

Figures 3A,B present HIVST average costs health facilities at program level by countries and distribution channel. They also highlight the proportion of site level costs ranging from 15 to 59%. In all countries, site level HIVST average costs range between $4 and 26 depending on the distribution channel and country (Table 3). Between 14 and 54% of these costs are induced by staff–Administration / management costs, 7–23% by staff-HIV testing providers, and 32–73% by HIVST kits costs. Average costs ranged from $4 to 8 in Côte d'Ivoire, $7 to 26 in Mali, and $4 to 5 in Senegal.

**Table 3:** Total and average site level HIV self-testing costs, by country, by distribution channel, and by expense item in health facilities (in 2021 US$).

	**Côte d'Ivoire**									
	**HIVST**									
	**STI**	**%**	**Index testing**	**%**	**MSM**	**%**	**FSW**	**%**	**PWUD**	**%**
Number of sites	10		12		3		3		1	
Personnel–Administration/management costs	1 431	*15%*	11 690	*45%*	2 314	*38%*	2 314	*40%*	3 583	*46%*
Personnel–HIV testing providers	1 280	*13%*	6 019	*23%*	875	*14%*	875	*15%*	1 263	*16%*
HIV self-testing kits	7 049	*72%*	8 278	*32%*	2 955	*48%*	2 670	*46%*	2 927	*38%*
Total annual costs	9 760		25 988		6 144		5 859		7 773	
HIVST kits distributed	2 782		3 260		1 128		1 019		1 117	
Average cost per HIVST kit distributed	4		8		6		6		7	
	**Mali**									
	**HIVST**									
	**STI**	**%**	**Index testing**	**%**	**MSM**	**%**	**FSW**	**%**		
Number of sites	5		8		4		4			
Personnel–Administration/management costs	2 613	*38%*	3 670	*38%*	2 622	*54%*	2 622	*39%*		
Personnel–HIV testing providers	1 339	*20%*	1 720	*18%*	669	*14%*	669	*10%*		
HIV self-testing kits	2 908	*42%*	4 226	*44%*	1 583	*32%*	3 469	*51%*		
Total annual costs	6 859		9 617		4 874		6 760			
HIVST kits distributed	948		1 378		516		1 131			
Average cost per HIVST kit distributed	25		16		26		7			
	**Senegal**									
	**HIVST**									
	**STI**	**%**	**Index testing**	**%**	**FSW**	**%**	**PWUD**	**%**		
Number of sites	5		3		1		1			
Personnel–Administration/management costs	1 132	*20%*	423	*22%*	282	*22%*	423	*14%*		
Personnel–HIV testing providers	379	*7%*	141	*7%*	94	*7%*	726	*23%*		
HIV self-testing kits	4 096	*73%*	1 372	*71%*	923	*71%*	1 969	*63%*		
Total annual costs	5 606		1 937		1 299		3 118			
HIVST kits distributed	1 331		451		300		640			
Average cost per HIVST kit distributed	4		4		4		5			

*HIVST, HIV Self-Testing kit; FSW, Female Sex workers; MSM, Men who have Sex with Men; PWUD, People who use drugs; STI, sexually transmitted infections; Index, sexual partners of PLHIV; PLHIV, people living with HIV*.

Site level HTS average costs per person tested are comparable between STI and Index Testing services and range from $7 to 8 (Appendix 4). The average costs per HIV diagnosis range from $72 to 705. The cost drivers are personnel costs representing between 65 and 71% of total costs across distribution channels and countries, followed by costs of supplies between 21 and 30% and all other costs between 2 and 13%.

### Sensitivity Analysis of Costs Results

Average observed costs per HIVST kit distributed remain robust when key cost parameters are varied (Appendix Figures 5A–C). In Côte d'Ivoire, varying life of start-up sensitization and training between 1 and 3 years has the strongest effect on costs ranging between $14–16, $14–15, $11–13, $29–31, $92–94 for FSW, MSM, PWUD, STI, and Index Testing respectively. The variation of the assumed length of life for the development and start-up phases, and the variation of the discount rate have a moderate effect with less than a dollar variation. Switching trained distributors with the size of PLHIV cohorts in sites has a small effect with <$2 variation of average costs in all countries. In Mali, the discount rate has the strongest effect for the Index Testing ($98–100), STIs ($124–126) and MSM ($89–91), but only varies by <$2 for other parameters.

## Discussion

In this study, based on routine and observed data from public health service providers and administrative partners, we estimated at program and site level the average costs of low-volume delivery models for implementing HIVST and HTS strategies in health facilities in three west African countries. To our knowledge, this is the first multi-country cost analysis of the integration of HIVST into health facilities with small dispensing volumes in west Africa.

HIVST average cost showed some variability between countries ranging from $12 to 93 in Côte d'Ivoire, $20 to 286 in Mali and $19 to 38 in Senegal. Site level HIVST average costs ranged $4–26 across distribution channels and countries. Site level HTS costs ranged $7–8 per testing session, and ranged $72–705 per HIV diagnosis. Across countries, average costs per HIVST were lowest for FSW, MSM and PWUD distribution channels followed by STI, then Index Testing distribution channel. These differences were mostly explained by the HIVST number of kits distributed between sites, so variation was observed between and within countries. Distribution channels with a lower volume of distribution had higher observed costs. Mali was marked by a very low dispensing volume for small sample health facilities with a total of dispensed 2326 kits for all distribution channels taken together but in Côte d'Ivoire, 2782 kits were distributed in STI services and 3260 kits for Index Testing over the same period of 18 months. Other factors also explained this. HIVST distribution for the Index Testing distribution channel is dependent on the PLHIV patients and sharing of their HIV status with their partner, a slow process that may require sensitization over several weeks/months of follow-up (25). It is also important to note that the different facility-based distribution channels could sometimes overlap as many individuals going to key population dedicated clinics are also STI patients and/or PLHIV.

HIVST small-scale health facility distribution channels are part of a national HIVST implementation program with large scale distribution channels for outreach HIVST generating high central costs (16, 26), so central costs are likely negligible for these low-scale distribution channels, when run alongside larger-scale models. In addition, central costs are also particularly high with the ATLAS integrated approach that entailed a lengthy implementation process with partners (16, 17), so even if our cost allocation method of central costs was based on volumes, these central costs would still remain high–as shown by our sensitivity analysis.

Such information is essential in designing sustainable and cost-effective models of HIVST as countries approach the UNAIDS 95-95-95 targets (21). In southern Africa, HIVST costing studies were conducted focusing either on outreach or on health facilities distribution of HIVST between 2017 and 2020. These studies reported average costs per kit distributed of $9.66 for Malawi, $17.70 for Zambia, and $14.91 for Zimbabwe and incremental average costs of $15.40 and 14.00 in early and later phases of an outreach HIVST distribution program in Lesotho (27, 28). Another health facility based HIVST distribution study was done in Malawi and reported average costs of $4.99 (21).

The average costs of the HIVST dispensation of the STI and Index Testing distribution channels, excluding central costs, are on the same scales or even lower than those of the HTS. However, cost comparison between HIVST and HTS should be cautious. In the case of Index Testing, HIVST provision is conducted through secondary distribution whereas HTS requires the partner to come to the health facility. When comparing with recent similar studies at health facilities in southern Africa, HIVST average cost through the primary distribution approach given by Sande and al. are $4.27 in Zambia and $9.24 in Zimbabwe (21), while Mwenge et al. found that the cost per individual person tested using HTS is $5.03 in Malawi, $4.24 in Zambia, and $8.79 in Zimbabwe (27).

A study was conducted by Ahmed et al. in low HIVST volume health facilities in Malawi, Zambia and Zimbabwe where costs ranged from US$5.20 to 58.92 per kit distributed. Higher costs were associated with low scale of activity and poor integration of HIVST distribution into existing health services (29). During the ATLAS project, it is likely that other factors played a role on the estimation of costs. For instance, In Mali, there were safety concerns due to the country's rebellion in August 2020 and ongoing armed conflict with an intermittent suspension of fieldwork activities. Finally, the COVID-19 pandemic also led to reduced activities during 2–3 months because of lockdown's restrictions in Côte d'Ivoire, Mali, and Senegal (30), leading to high observed costs, although self-testing was shown to be a timely alternative to provider-delivered HIV testing during periods of lockdown and reduced social interactions.

Across countries, the site level average cost of integrating HIVST into health facilities ranged from $4 to 26 per kit distributed. Site level costs would be considered closer to those used for financial planning than program level average costs. Those per HTS carried out varied between $7–8. Personnel and HIVST kits were the major cost drivers. So, the average costs of introducing HIVST in fixed low-volume strategies were slightly higher than those of HTS. These costs being comparable, there is an opportunity to diversify the offer of HIV testing at health facilities and potentially improve access to screening for target populations not reached by HTS. Consideration of task shifting for the HIVST distribution through Index testing distribution channels from medical team to non-medical could also be cost saving as it was suggested with a case study in Mali in 2020 (17).

This study has some limitations. First, our outcome metric “per HIVST kit distributed” does not fully capture the HIVST cascade and additional costs would be incurred for linkage to confirmatory testing and HIV care services. For example, there remain uncertainties related to the true percentage of kits used in the secondary distribution model. Second, total and average costs are estimated across a diverse range of fixed health facilities for each country leading to inevitable cost variation by distribution channel. Third, reduced sample sizes from original targets were due to COVID restrictions for data collection and challenging partnerships with public sites, particularly in Mali. Therefore, results for this country should be interpreted with caution. Finally, cost analyses of small-scale interventions often generate extreme average costs per output without much application for program planning. Our suggestion to consider site level costs for financial planning in this specific study assumes that HIVST programs in these countries would always have larger-scale mobile HIVST distribution strategies in place where central costs would be accounted for, which is the case of the ATLAS project. However, the risk of underbudgeting such small-scale fixed strategies by cutting off too much central costs still remain and requires careful financial planning.

Ultimately, economic valuation of real-world costs from small-volume health facilities can offer valuable information to improve future program operations and should therefore be included as an important element in overall program planning (31). Although the average costs of HIVST at the program level were high, the average costs of HIVST at the site level remained comparable to the costs of HTS in all countries. Low-volume, facility-based distribution channels have high core costs that need to be carefully considered for budgeting of small volume health interventions. HIVST can diversify the offer of HIV testing in health facilities and would allow to expand the range of screening services to target populations difficult to reach with HTS services while identifying “best practices” that can inform the successful implementation of programs on a global scale.

## Data Availability Statement

The raw data supporting the conclusions of this article will be made available by the authors, without undue reservation.

## Ethics Statement

The studies involving human participants were reviewed and approved by the London School of Hygiene and Tropical Medicine Research Committee (n° 17141/RR/13198, 31st March 2019) WHO Ethic Research Committee (n°ERC0003181, 7th August 2019), and by three national ethic committees: Comité National d'Ethique des Sciences de la vie et de la Santé de Côte d'Ivoire (n°049-19/MSHP/CNESVS-kp, 28th May 2019), ComitéNational d'Ethique pour la Recherche en santé du Sénégal (n°SEN19/32, 26th July 2019), and Comité d'Ethique de la Faculté de Médecine de Pharmacie et d'Odonto-Stomatologie de l'Université des Sciences et des Techniques de Bamako au Mali (n°2019/88/CE/FMPOS, 14th August 2019). The patients/participants provided their written informed consent to participate in this study.

## Author Contributions

Md'E and FT-P designed the study. Md'E coordinated the study. MT and KB conducted data collection and analysis and wrote the article. AV, AS, OK, NR, MM-G, M-CB, and JL provided logistical support and intellectual inputs. All authors revised the manuscript and agreed for publication.

## Funding

The study was funded by UNITAID through Solthis. This work was supported by UNITAID (Grant Number 2018-23-ATLAS) with additional funding from Agence Française pour le Développement (AFD). The funders played no role in study design, data collection and analysis, the decision to publish or preparation of the manuscript.

## Author Disclaimer

The content is solely the responsibility of the authors and does not necessarily represent the official views of UNITAID, Solthis, or UNAIDS.

## Conflict of Interest

The authors declare that the research was conducted in the absence of any commercial or financial relationships that could be construed as a potential conflict of interest.

## Publisher's Note

All claims expressed in this article are solely those of the authors and do not necessarily represent those of their affiliated organizations, or those of the publisher, the editors and the reviewers. Any product that may be evaluated in this article, or claim that may be made by its manufacturer, is not guaranteed or endorsed by the publisher.

## Figures and Tables

**Table:** 

	**ATLAS Research Team**
Amani Elvis Georges	Programme PACCI, ANRS Research Site, Treichville University Hospital, Abidjan, Côte d'Ivoire.
Badiane Kéba	Solthis, Sénégal
Bayac Céline	Solthis, France
Bekelynck Anne	Programme PACCI, ANRS Research Site, Treichville University Hospital, Abidjan, Côte d'Ivoire
Boily Marie-Claude	Medical Research Council Centre for Global Infectious Disease Analysis, Department of Infectious Disease Epidemiology, Imperial College London, London, United Kingdom
Boye Sokhna	Centre Population et Développement, Institut de Recherche pour le Développement, Université Paris Descartes, Inserm, Paris, France
Breton Guillaume	Solthis, Paris, France
d'Elbée Marc	Department of Global Health in the Global South, Bordeaux Population Health, Institut national de la santé et de la recherche médicale (Inserm), University of Bordeaux (France)
Desclaux Alice	Institut de Recherche pour le Développement, Transvihmi (UMI 233 IRD, 1175 INSERM, Montpellier University), Montpellier, France/CRCF, Dakar, Sénégal
Desgrées du Loû Annabel	Centre Population et Développement, Institut de Recherche pour le Développement, Université Paris Descartes, Inserm, Paris, France
Ehui Eboi	Directeur Coordonnateur, PNLS
Graham Medley	Department of Global Health and Development, Faculty of Public Health and Policy, London School of Hygiene and Tropical Medicine, London, UK
Jean Kévin	Laboratoire MESuRS, Conservatoire National des Arts et Métiers, Paris, France
Keita Abdelaye	Institut National de Recherche en Santé Publique, Bamako, Mali
Kouassi Kra Arsène	Centre Population et Développement, Institut de Recherche pour le Développement, Université Paris Descartes, Inserm, Paris, France
Ky-Zerbo Odette	TransVIHMI, IRD, Université de Montpellier, INSERM
Larmarange Joseph	Centre Population et Développement, Institut de Recherche pour le Développement, Université Paris Descartes, Inserm, Paris, France
Maheu-Giroux Mathieu	Department of Epidemiology, Biostatistics, and Occupational Health, School of Population and Global Health, McGill University, Montréal, QC, H3A 1A2, Canada
Moh Raoul	1. Programme PACCI, ANRS Research Site, Treichville University Hospital, Abidjan, Côte d'Ivoire. 2. Department of Infectious and Tropical Diseases, Treichville University Teaching Hospital, Abidjan, Côte d'Ivoire. 3. Medical School, University Felix Houphouet Boigny, Abidjan, Côte d'Ivoire
Mosso Rosine	ENSEA Ecole Nationale de Statistiques et d'Economie Appliquée, Abidjan, Côte d'Ivoire
Ndour Cheikh Tidiane	Division de Lutte contre le Sida et les IST, Ministère de la Santé et de l'Action Sociale Institut d'Hygiène Sociale, Dakar, Sénégal
Paltiel David	Yale School of Public Health, New Haven, CT, USA
Pourette Dolorès	Centre Population et Développement, Institut de Recherche pour le Développement, Université Paris Descartes, Inserm, Paris, France
Rouveau Nicolas	Centre Population et Développement, Institut de Recherche pour le Développement, Université Paris Descartes, Inserm, Paris, France
Silhol Romain	Medical Research Council Centre for Global Infectious Disease Analysis, Department of Infectious Disease Epidemiology, Imperial College London, London, United Kingdom
Simo Fotso Arlette	Centre Population et Développement, Institut de Recherche pour le Développement, Université Paris Descartes, Inserm, Paris, France
Terris-Prestholt Fern	Department of Global Health and Development, Faculty of Public Health and Policy, London School of Hygiene and Tropical Medicine, London, United Kingdom
Traore Métogara Mohamed	Solthis, Côte d'Ivoire
	**Solthis coordination team**
Diop Papa Moussa	Solthis, Sénégal
Doumenc-Aïdara Clémence	Solthis, Dakar, Sénégal
Geoffroy Olivier	Solthis, Abidjan, Côte d'Ivoire
Kabemba Odé Kanku	Solthis, Bamako, Mali
Papa Alioune Gueye	Solthis, Dakar, Sénégal
Vautier Anthony	Solthis, Dakar, Sénégal
	**Implementation in Côte d'Ivoire**
Abokon Armand	Fondation Ariel Glaser, Côte d'Ivoire
Anoma Camille	Espace Confiance, Côte d'Ivoire
Diokouri Annie	Fondation Ariel Glaser, Côte d'Ivoire
Kouame Blaise	Service Dépistage, PNLS
Kouakou Venance	Heartland Alliance, Côte d'Ivoire
Koffi Odette	Aprosam, Côte d'Ivoire
Kpolo Alain-Michel	Ruban Rouge, Côte d'Ivoire
Tety Josiane	Blety, Côte d'Ivoire
Traore Yacouba	Orasur, Côte d'Ivoire
	**Implementation in Mali**
Bagendabanga Jules	FHI 360, Mali
Berthé Djelika	PSI, Mali
Diakite Daouda	Secrétariat Exécutif du Haut Conseil National de Lutte contre le Sida, Mali
Diakité Mahamadou	Danayaso, Mali
Diallo Youssouf	CSLS/MSHP
Daouda Minta	Comité scientifique VIH
Hessou Septime	Plan Mali
Kanambaye Saidou	PSI, Mali
Kanoute Abdul Karim	Plan Mali
Keita Dembele Bintou	Arcad-Sida, Mali
Koné Dramane	Secrétariat Exécutif du Haut Conseil National de Lutte contre le Sida, Mali
Koné Mariam	AKS, Mali
Maiga Almoustapha	Comité scientifique VIH
Nouhoum Telly	CSLS/MSHP
Saran Keita Aminata	Soutoura, Mali
Sidibé Fadiala	Soutoura, Mali
Tall Madani	FHI 360, Mali
Yattassaye Camara Adam	Arcad-Sida, Mali
Sanogo Abdoulaye	Amprode Sahel, Mali
	**Implementation in Senegal**
Bâ Idrissa	CEPIAD, Sénégal
Diallo Papa Amadou Niang	CNLS, Sénégal
Fall Fatou	DLSI, Ministère de la Santé et de l'action sociale, Sénégal
Guèye NDèye Fatou NGom	CTA, Sénégal
Ndiaye Sidy Mokhtar	Enda Santé, Sénégal
Niang Alassane Moussa	DLSI, Ministère de la Santé et de l'action sociale, Sénégal
Samba Oumar	CEPIAD, Sénégal
Thiam Safiatou	CNLS, Sénégal
Turpin Nguissali M.E.	Enda Santé, Sénégal
	**Partners**
Bouaré Seydou	Assistant de recherche, Mali
Camara Cheick Sidi	Assistant de recherche, Mali
Kouadio Brou Alexis	Assistant de recherche, Côte d'Ivoire
Sarrassat Sophie	Centre for Maternal, Adolescent, Reproductive and Child Health, London School of Hygiene and Tropical Medicine, London, UK
Sow Souleymane	Assistant de recherche, Sénégal
