# Supplementary material for: Economic Analysis of Low Volume Interventions Using Real-World Data: Costs of HIV Self-Testing Distribution and HIV Testing Services in West Africa From the ATLAS Project
Source: Front Health Serv. 2022 Jun 27;2:886513. doi: 10.3389/frhs.2022.886513 (PMC10012764; doi:10.3389/frhs.2022.886513)
Supplement: Supplementary file 1 [file Data_Sheet_1.PDF]

Supplementary Material

Appendix 1 ATLAS HIVST delivery channels

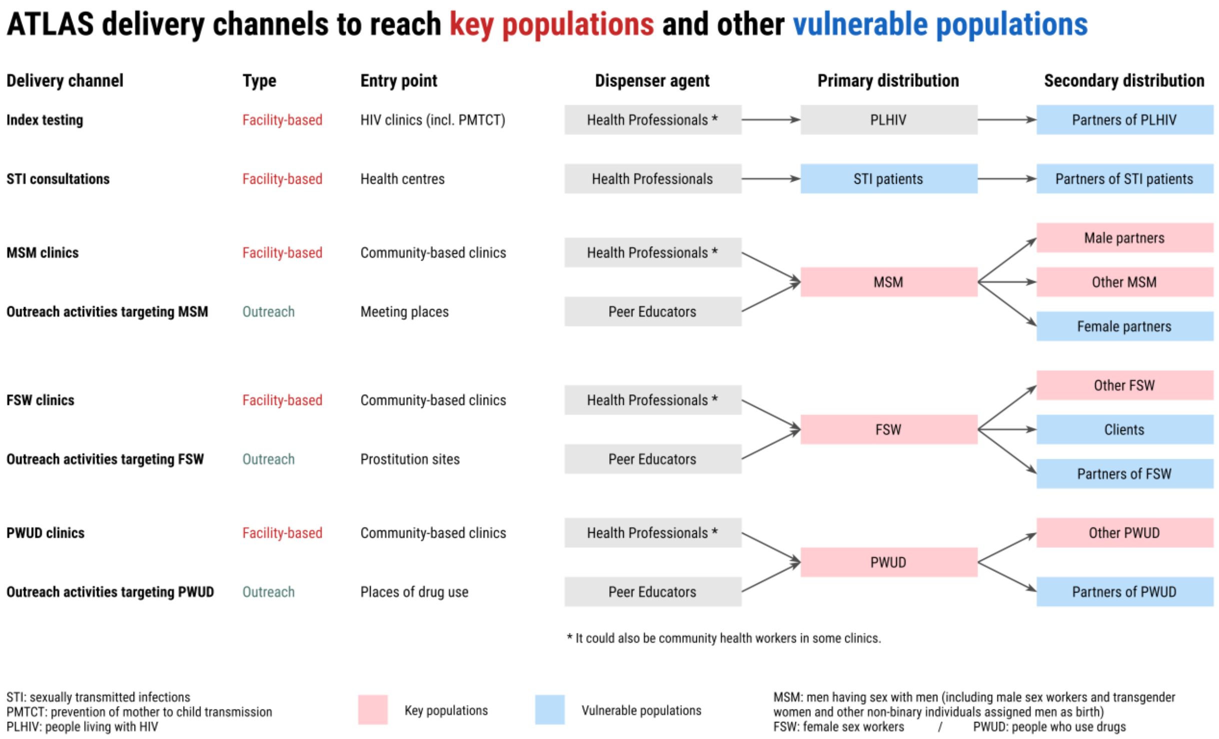

Appendix 2 Sources of cost inputs and of cost allocation data for the top-down (TD) and bottom-up (BU) costing approaches using a provider's perspective (costs at site level in grey, costs above site level in white)

**Testing services**	HIVST						HTS	
**Intervention phases**	Development		Start up		Implementation		Implementation	
Costing approach	TD	BU	TD	BU	TD	BU	TD	BU
**Capital**								
Building & storage	x		x		x	x		x
Equipment	x		x		x	x		x
Vehicles	x		x		x			
Other capital costs	x		x		x			
**Recurrent**								
Training			x					
Sensitization – Coordination			x					
Sensitization – Solthis pays			x					
Personnel & Per diems Headquarters Solthis Pays	x		x		x			
Personnel & Per diems – Coordination	x		x		x			
Personnel & Per diems - Headquarters (implementing partners)					x	x		x
Personnel & Per diems – Field (HIVST distributors)					x	x		x
HIV self-testing kits/HTS session					x			x
Vehicle operation, maintenance & transport	x		x		x			x
Building operation/maintenance	x		x		x			x
Other recurrent	x		x		x			x

Appendix 3 Allocation factors for the top-down costing analysis by input type

	Allocation factors to site level		
**Input type**	**Côte d'Ivoire**	**Senegal**	**Mali**
Start-up costs			
S1. Trainings	% trained distributors	% trained distributors	% trained distributors
S2. Sensitization	% of cohort size	% of cohort size	% of cohort size
Capital costs			
C1. Buildings and storage	% direct expenditure	% direct expenditure	% direct expenditure
C2. Equipment	% direct expenditure	% direct expenditure	% direct expenditure
C3. Vehicles	% HIVST kits distributed	% HIVST kits distributed	% HIVST kits distributed
C4. Other capital costs	% direct expenditure	% direct expenditure	% direct expenditure
Recurrent costs			
R1. Personnel & Per diems – Headquarters IPO coordination	% trained distributors	Equally shared across sites	% trained distributors
R2. Personnel & Per diems – Headquarters IPO country	% trained distributors	Equally shared across sites	% trained distributors
R3. Personnel & Per diems – Headquarters Implementing partner	% trained distributors	% HIVST distributors	% trained distributors
R4. Personnel & Per diems – Field - HIVST distributors	% trained distributors	% HIVST distributors	% trained distributors
R5. HIV self-testing kits	% HIVST kits distributed	% HIVST kits distributed	% HIVST kits distributed
R6. Vehicle operation and maintenance/transportation	% HIVST kits distributed	% HIVST kits distributed	% HIVST kits distributed
R7. Building operation and maintenance	% direct expenditure	% direct expenditure	% direct expenditure
R8. Other recurrent costs	% direct expenditure	% direct expenditure	% direct expenditure

Appendix 4 Total, testing sessions, Average cost per HTS session, Average cost per HTS-positive identified of HTS costs, by country, by channel, and by expense item in health facilities (in 2021 US$)

	Côte d'Ivoire HTS				Mali HTS		Senegal HTS			
	**STI**	**%**	**Index testing**		**STI/Index testing**	**%**	**STI**	**%**	**Index testing**	**%**
**Number of sites**	**10**		**12**		**6**		**5**		**4**	
**Capital costs**										
Buildings and storage	64	*1%*	68	*1%*	727	*1%*	1168	*4%*	1224	*2%*
Equipment	7	*0%*	8	*0%*	73	*0%*	117	*0%*	122	*0%*
**Recurrent Costs**										
Personnel – Administration/management costs	841	*11 %*	2 909	*23 %*	30784	*27 %*	5183	*18%*	17872	*30 %*
Personnel – HIV testing providers	4 138	*56 %*	5 929	*48 %*	44675	*39 %*	13540	*47%*	24457	*41 %*
Testing supplies	2 211	*30 %*	3 326	*27 %*	28632	*25 %*	6113	*21%*	14566	*24 %*
Vehicle operation and maintenance	10	*0%*	39	*0%*	2480	*2%*	0	*0%*	0	*0%*
Building operation and maintenance	45	*1%*	57	*0%*	4816	*4%*	1738	*6%*	733	*1%*
Other recurrent costs	82	*1%*	88	*1%*	1086	*1%*	926	*3%*	886	*1%*
**TOTAL COSTS**	7 397		12 423		113273		28784		59861	
**HTS testing sessions (HIV diagnosis)**	1073 (02)		1613 (148)		18839 (2118)		3929 (42)		9385 (89)	
**Average cost per HTS session (min-max)**	8 (6-10)		8 (6-14)		8 (4-14)		8 (3-11)		7 (3-11)	
**Average cost per HIV diagnosis (min-max)**	*185 (161-209)		*87 (40-145)		72 (21-147)		705 (364-1032)		662 (301-854)	

HTS: traditional HIV testing services; STI: partners of sexually transmitted infections patients; Index Testing: sexual partners of PLHIV; PLHIV people living with HIV
**For the cost per HIV diagnosis in Côte d'Ivoire, some sites did not identify HIV-positive cases during the observation period through STI (8/10 sites) and Index testing (10/12 sites) channels and were excluded from the analysis*

Appendix 5a Tornado diagrams of findings from deterministic sensitivity analysis in health facilities in Côte d'Ivoire

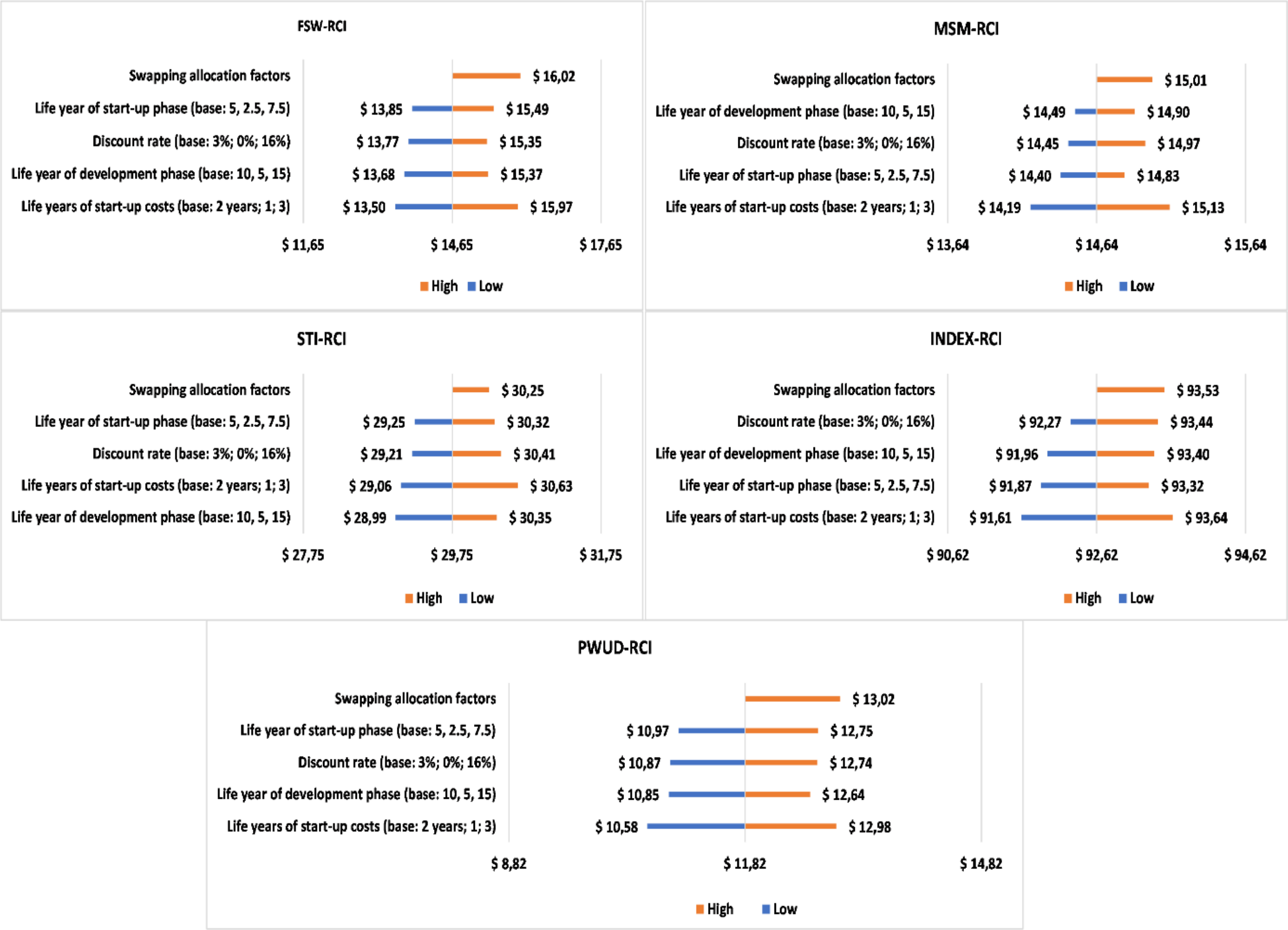

HIVST: HIV Self-Testing kit; HTS: HIV Testing Services; FSW: Female Sex workers; MSM: Men who have Sex with Men; PWUD: People who use drugs; STI: STI: partners of sexually transmitted infections patients; PLHIV people living with HIV; Index Testing: sexual partners of PLHIV;

Appendix 5b Tornado diagrams of findings from deterministic sensitivity analysis in health facilities in Mali

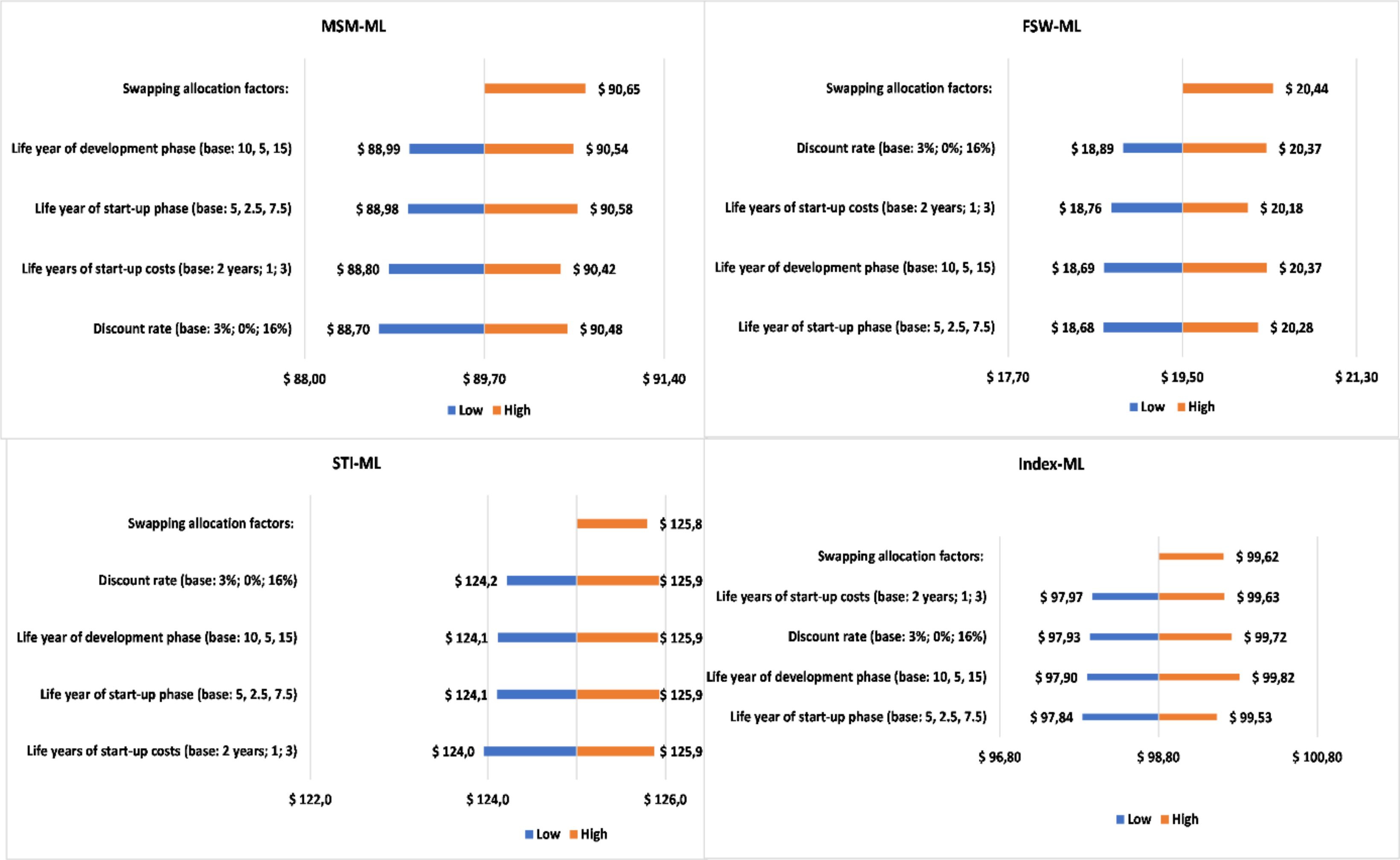

HIVST: HIV Self-Testing kit; HTS: HIV Testing Services; FSW: Female Sex workers; MSM: Men who have Sex with Men; PWUD: People who use drugs; STI: STI: partners of sexually transmitted infections patients; PLHIV people living with HIV; Index Testing: sexual partners of PLHIV;

Appendix 5c Tornado diagrams of findings from deterministic sensitivity analysis in health facilities in Senegal

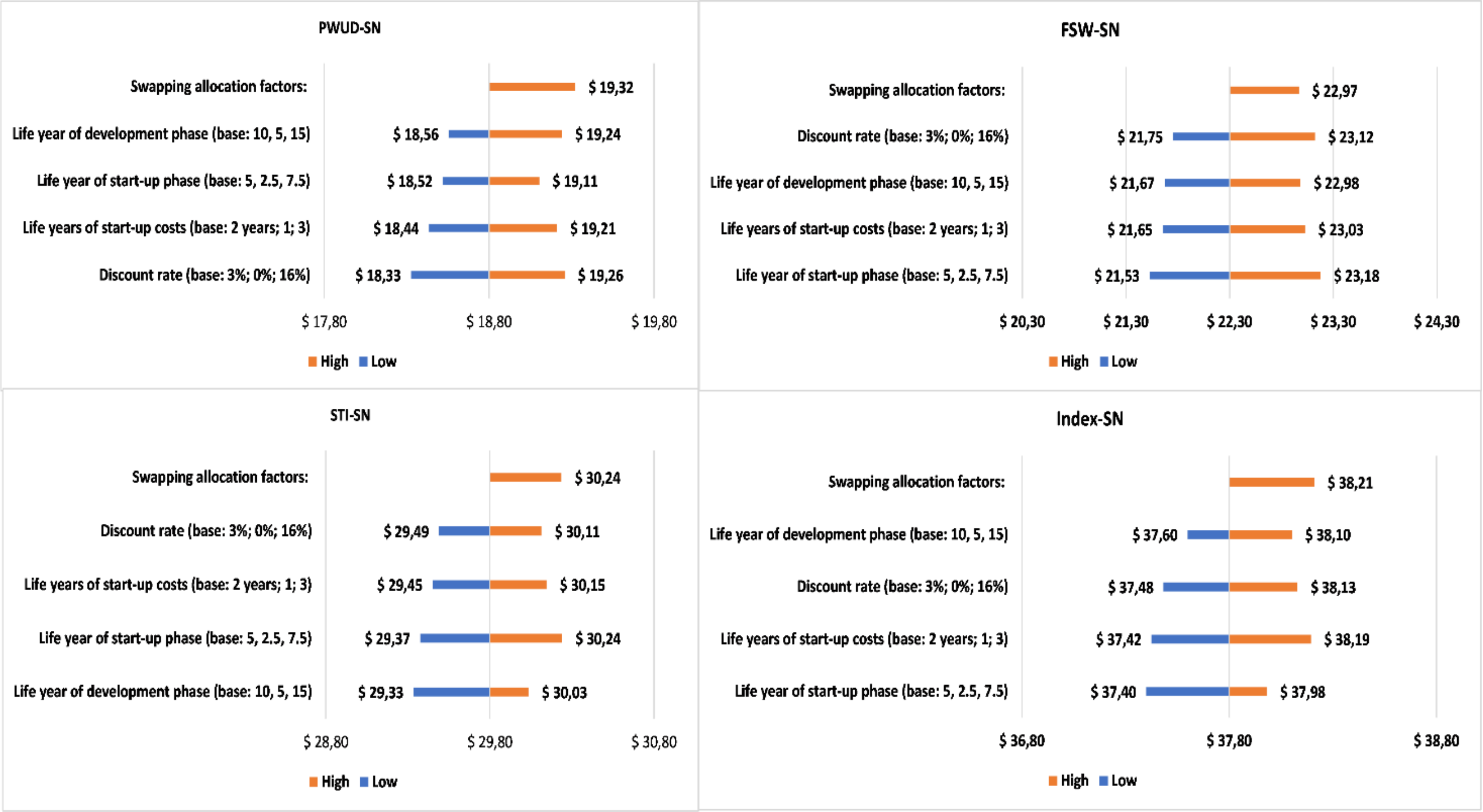

HIVST: HIV Self-Testing kit; HTS: HIV Testing Services; FSW: Female Sex workers; MSM: Men who have Sex with Men; PWUD: People who use drugs; STI: STI: partners of sexually transmitted infections patients; PLHIV people living with HIV; Index Testing: sexual partners of PLHIV;
